# Theta-frequency selectivity in the somatic spike-triggered average of rat hippocampal pyramidal neurons is dependent on HCN channels

**DOI:** 10.1152/jn.00356.2017

**Published:** 2017-08-02

**Authors:** Anindita Das, Rishikesh Narayanan

**Affiliations:** Cellular Neurophysiology Laboratory, Molecular Biophysics Unit, Indian Institute of Science, Bangalore, India

**Keywords:** coincidence detection, electrophysiology, frequency selectivity, HCN channels, hippocampus, spike initiation dynamics, spike-triggered average

## Abstract

The ability to distill specific frequencies from complex spatiotemporal patterns of afferent inputs is a pivotal functional requirement for neurons residing in networks receiving frequency-multiplexed inputs. Although the expression of theta-frequency subthreshold resonance is established in hippocampal pyramidal neurons, it is not known if their spike initiation dynamics manifest spectral selectivity, or if their intrinsic properties are tuned to process gamma-frequency inputs. Here, we measured the spike-triggered average (STA) of rat hippocampal pyramidal neurons through electrophysiological recordings and quantified spectral selectivity in their spike initiation dynamics and their coincidence detection window (CDW). Our results revealed strong theta-frequency selectivity in the STA, which was also endowed with gamma-range CDW, with prominent neuron-to-neuron variability that manifested distinct pairwise dissociations and correlations with different intrinsic measurements. Furthermore, we demonstrate that the STA and its measurements substantially adapted to the state of the neuron defined by its membrane potential and to the statistics of its afferent inputs. Finally, we tested the effect of pharmacologically blocking the hyperpolarization-activated cyclic-nucleotide-gated (HCN) channels on the STA and found that the STA characteristic frequency reduced significantly to the delta-frequency band after HCN channel blockade. This delta-frequency selectivity in the STA emerged in the absence of subthreshold resonance, which was abolished by HCN channel blockade, thereby confirming computational predictions on the dissociation between these two forms of spectral selectivity. Our results expand the roles of HCN channels to theta-frequency selectivity in the spike initiation dynamics, apart from underscoring the critical role of interactions among different ion channels in regulating neuronal physiology.

**NEW & NOTEWORTHY** We had previously predicted, using computational analyses, that the spike-triggered average (STA) of hippocampal neurons would exhibit theta-frequency (4–10 Hz) spectral selectivity and would manifest coincidence detection capabilities for inputs in the gamma-frequency band (25–150 Hz). Here, we confirmed these predictions through direct electrophysiological recordings of STA from rat CA1 pyramidal neurons and demonstrate that blocking HCN channels reduces the frequency of STA spectral selectivity to the delta-frequency range (0.5–4 Hz).

## Abstract (precis)

We had previously predicted, using computational analyses, that the spike-triggered average (STA) of hippocampal neurons would exhibit theta-frequency (4–10 Hz) spectral selectivity and would manifest coincidence detection capabilities for inputs in the gamma-frequency band (25–150 Hz). Here, we confirmed these predictions through direct electrophysiological recordings of STA from rat CA1 pyramidal neurons and demonstrate that blocking HCN channels reduces the frequency of STA spectral selectivity to the delta-frequency range (0.5–4 Hz).

the spike-triggered average (STA) quantifies the linear response filters of single neurons and proffers a description of neuronal spike initiation dynamics, lending it as a useful tool to link neuronal dynamics at the network scale to intrinsic feature selectivity of single neurons. The STA is typically computed by averaging inputs that trigger spikes when neuronal compartments are injected with white noise current inputs and, therefore, constitutes a direct unbiased measure of the dynamic structure of inputs that initiate action potentials in the neuron under consideration. The STA has been shown to be critically dependent on the neuronal ion channels and intrinsic properties, the state of neuromodulatory afferents, as well as the nature of input that is being processed (Agüera y Arcas and Fairhall 2003; Badel et al. 2008; Bryant and Segundo 1976; Das et al. 2017; Das and Narayanan 2014, 2015; Dayan and Abbott 2005; Eggermont et al. 1983; Ermentrout et al. 2007; Famulare and Fairhall 2010; Haas et al. 2007; Mainen and Sejnowski 1995; Prescott et al. 2008a; Ratté et al. 2013; Rieke et al. 1999; Schwartz et al. 2006).

In assessing the relationship between the STA, neuronal feature selectivity, and coincidence detection, recent studies have derived STA-based quantitative metrics that could be used to assess neuronal suprathreshold frequency selectivity and coincidence detection window (CDW). These computational studies demonstrated a clear dependence of STA-based metrics on channel expression profiles, with specific channels capable of effectuating transitions that span the integrator-coincidence detector continuum characterized by the class of STA (Das et al. 2017; Das and Narayanan 2014, 2015). Specifically, the expression of specific channels has been shown to mediate transitions of neurons or compartments between class I (integrators) and class II/III (coincidence detectors) excitability. Although classes of excitability were defined by Hodgkin (1948) based on differences in the firing rate profiles obtained with different current injections, this classification has been extremely helpful in understanding several aspects of neuronal and network physiology, including coincidence detection, feature selectivity, and network synchrony (Abouzeid and Ermentrout 2009; Das et al. 2017; Das and Narayanan 2014, 2015; Ermentrout 1996; Ermentrout et al. 2007; Golding and Oertel 2012; Khurana et al. 2011, 2012; Llinas et al. 2002; Mathews et al. 2010; Padmanabhan and Urban 2010; Prescott et al. 2008a; Ratté et al. 2013).

With reference to hippocampal pyramidal neurons, computational studies have predicted a prominent role for the hyperpolarization-activated cyclic nucleotide gated (HCN) channels in mediating theta-frequency (4–10 Hz) spectral selectivity and gamma-range (slow: 25–60 Hz and fast: 60–150 Hz) coincidence detection (Das et al. 2017; Das and Narayanan 2014, 2015). The HCN channels, also known as the pacemaker channels, are slow channels that are activated by hyperpolarization and express heavily in hippocampal pyramidal neurons and their dendrites (Biel et al. 2009; Gasparini and DiFrancesco 1997; He et al. 2014; Lörincz et al. 2002; Magee 1998; Pape 1996; Robinson and Siegelbaum 2003; Shah 2014). HCN channels have been shown to play several critical neurophysiological roles in regulating resting membrane potential (RMP) (Gasparini and DiFrancesco 1997; Magee 1998; Mishra and Narayanan 2015; Poolos et al. 2002), neuronal excitability (Brager and Johnston 2007; Fan et al. 2005; Gasparini and DiFrancesco 1997; Magee 1998; Mishra and Narayanan 2015; Narayanan et al. 2010; Narayanan and Johnston 2007; Poolos et al. 2002; van Welie et al. 2004), temporal summation (Magee 1998, 1999b, 2000; Williams and Stuart 2000), subthreshold resonance (Hu et al. 2009, 2002; Hutcheon et al. 1996a, 1996b; Hutcheon and Yarom 2000; Narayanan and Johnston 2007), neuronal oscillations (Dickson et al. 2000; Fransén et al. 2004; Lüthi and McCormick 1998a, 1998b), somatodendritic coupling (Cook et al. 2007; Hu et al. 2009; Ulrich 2002; Vaidya and Johnston 2013), intrinsic phase response (Marcelin et al. 2009; Narayanan and Johnston 2008; Rathour and Narayanan 2012, 2014; Vaidya and Johnston 2013), synaptic plasticity profiles (Anirudhan and Narayanan 2015; Honnuraiah and Narayanan 2013; Narayanan and Johnston 2010; Nolan et al. 2004), local field potentials (LFPs) (Ness et al. 2016; Sinha and Narayanan 2015), and neuronal spike phases with reference to external oscillations and their coherence (Sinha and Narayanan 2015).

In this study, we directly tested computational predictions from previous studies (Das et al. 2017; Das and Narayanan 2014, 2015), on the quantitative aspects of the STA of hippocampal pyramidal neurons and on the role of HCN channels in quantitatively altering specific STA characteristics, using patch-clamp electrophysiology. We injected Gaussian white noise (GWN) current into the neuronal somata and computed the STA as the average of specific stimuli that preceded the generation of action potentials. We assessed five different STA measurements defining spectral selectivity and coincidence detection (Das et al. 2017; Das and Narayanan 2014, 2015), and demonstrated theta-band spectral selectivity and gamma-range coincidence detection in the somatic STA of rat hippocampal pyramidal neurons. Employing multiple measurements of STA from the same neurons, we then demonstrated that STA and its measurements exhibited significant adaptability to membrane voltage and input statistics. Finally, we computed STA and other intrinsic properties in the presence of a pharmacological agent that blocked HCN channels and found theta-frequency selectivity in the STA to be critically reliant on HCN channels. Our results expand the roles of HCN channels to theta-frequency selectivity in the spike initiation dynamics, thereby emphasizing a critical role for these channels in neural coding and in defining the position of a neuron along the integrator-coincidence detector continuum of neuronal excitability.

## MATERIALS AND METHODS

### 

#### Ethical approval.

All experiments reported in this study were performed in strict adherence to the protocols cleared by the Institute Animal Ethics Committee (IAEC) of the Indian Institute of Science, Bangalore. Surgical and electrophysiological procedures were similar to previously established protocols (Ashhad et al. 2015; Ashhad and Narayanan 2016; Rathour et al. 2016) and are detailed below.

#### Surgery and slice preparation.

Five- to 10-week-old male Sprague-Dawley rats were anesthetized by intraperitoneal injection of a combination of ketamine and xylazine, and onset of deep anesthesia was determined by cessation of toe-pinch reflex. Rats were transcardially perfused with ice-cold cutting solution containing the following (in mM): 210 sucrose, 2.5 KCl, 1.25 NaH_2_PO_4_, 25 NaHCO_3_, 0.5 CaCl_2_, 7 MgCl_2_, 7 dextrose, and 3 sodium pyruvate (Sigma Aldrich). The animals were then decapitated, and the brain was removed quickly in the presence of ice-cold cutting solution. Near-horizontal middle (bregma –6.5 mm to –5.1 mm) hippocampal slices (350 µm) were prepared, using DTK Microslicer Zero 1 (Ted Pella), while submerged in oxygenated ice-cold cutting solution. The slices were incubated for 15–20 min at 34°C in a holding chamber containing the following (in mM): 125 NaCl, 2.5 KCl, 1.25 NaH_2_PO_4_, 25 NaHCO_3_, 2 CaCl_2_, 2 MgCl_2_, 10 dextrose, and 3 sodium pyruvate and then at room temperature for 1 h before recording. The holding chamber was continuously carbogenated with a mixture of 95% O_2_ and 5% CO_2_ gas.

#### Electrophysiology.

**Fig. 1.**
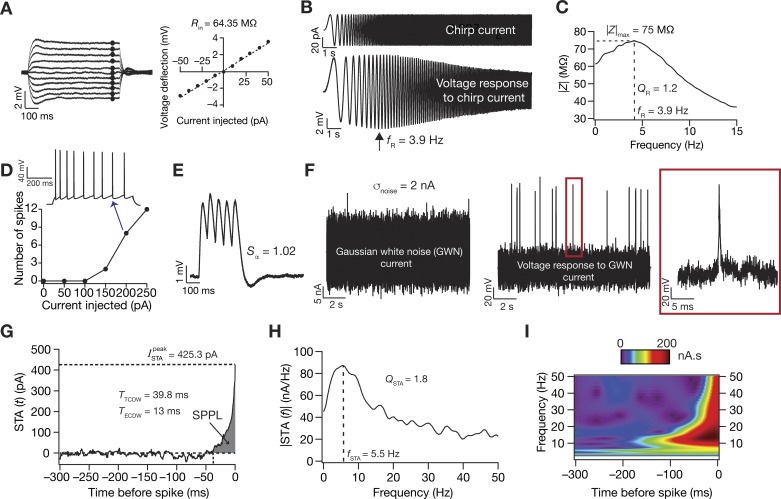
Traces from a typical experiment showing the different electrophysiological measurements. *A*: input resistance (*R*_in_) was measured by fitting a straight line to a steady-state *I–V* curve obtained from injecting long (700 ms) current pulses with amplitude −50 to +50 pA. *B*: resonance frequency (*f*_R_), resonance strength (*Q*_R_), and maximum impedance amplitude (|*Z*|_max_) were measured from the neuronal response to a chirp current input. *C*: the impedance amplitude profile was derived from the neuronal response to calculate these measurements. *D*: firing rate profile was obtained by injecting pulse currents of various amplitudes for 700 ms. An example trace of the voltage response to a pulse current injection of 200 pA showing spike frequency adaptation in firing rate is also shown. *E*: α-excitatory postsynaptic potential (EPSP) summation ratio (*S*_α_) was computed as the ratio of the last to the first α-EPSP amplitude in a train with a frequency of 20 Hz. *F*–*H*: Gaussian white noise (GWN) with a standard deviation adjusted to obtain ~1-Hz firing was injected into the neuron (*F*), and multiple sweeps were recorded to obtain ~1,000 spikes for computing the STA (*G*). STA measures included the peak STA current ($I_{\text{STA}}^{\text{peak}}$), the total and effective coincidence detection windows (*T*_TCDW_ and *T*_ECDW_, respectively), STA characteristic frequency (*f*_STA_), and selectivity strength (*Q*_STA_). *f*_STA_ and *Q*_STA_ were computed from the Fourier transformed version of STA (*H*). The shaded region in *G* represents the spike proximal positive lobe (SPPL), which was employed in the computation of *T*_ECDW_ (*Eq. 1*). *I*: time-frequency representation of the STA derived from a Morlet-wavelet transformed version of the STA shows selectivity that is largely confined to the spike-proximal 100 ms.

Slices were visualized under ×63 water immersion lens through a Dodt contrast microscope (Carl Zeiss Axioexaminer). Somatic whole cell current-clamp recordings were made from CA1 pyramidal neurons using a Dagan BVC-700A amplifier. Multiple steps were taken to ensure that the recorded cells were indeed CA1 pyramidal neurons. First, the pyramidal cell layer (stratum pyramidale) was visually identified, and neurons with pyramidal somatic morphology with their soma within the stratum pyramidale were selected. The apical dendritic arbor was followed to ensure visually that the dendritic trunk spread at least to the distal stratum radiatum. These morphological identification procedures allowed us to avoid interneurons, which do not have large apical dendritic trunks and pyramidal-like principal cells whose cell bodies are present above the stratum pyramidale, in the stratum radiatum (Bullis et al. 2007). Second, we compared a range of electrophysiological measurements (Fig. 1, *A–E*), which were computed online as the recordings were being performed, to existing electrophysiological characterization of CA1 pyramidal neurons from our laboratory and others (Ashhad et al. 2015; Ashhad and Narayanan 2016; Dougherty et al. 2012, 2013; Malik et al. 2016; Narayanan et al. 2010; Narayanan and Johnston 2007; 2008; Rathour et al. 2016; Rathour and Narayanan 2012). The presence of specific combinations of electrophysiological signatures spanning all of these measurements allowed us to unambiguously identify CA1 pyramidal neurons.

Data acquisition was done using custom-written software (default sampling rate: 40 kHz) in Igor Pro environment (Wavemetrics). During the entire course of experiments, the slices were perfused with carbogenated artificial cerebrospinal fluid heated through an inline heater (Warner Instruments) to ~34°C containing the following (in mM): 125 NaCl, 3 KCl, 1.25 NaH_2_PO_4_, 25 NaHCO_3_, 2 CaCl_2_, 1 MgCl_2_, and 10 dextrose. All experiments were performed in the presence of synaptic blockers (in µM): 10 6-cyano-7-nitroquinoxaline-2,3-dione (CNQX), 10 (+)bicuculline, 10 picrotoxin, and 2 (2*S*)-3-[[(1*S*)-1-(3,4-dichlorophenyl) ethyl] amino-2-hydroxypropyl] (phenylmethyl) phosphinic acid (CGP55845; Allied Scientific). Borosilicate glass electrodes, pulled (P-97 Flaming/Brown puller, Sutter) from capillaries of 1.5-mm outer diameter and 0.86-mm inner diameter (Sutter), of 4–7 MΩ resistance were used for recordings. The intracellular pipette solution contained the following (in mM): 120 potassium-gluconate, 20 KCl, 10 HEPES, 4 NaCl, 4 MgATP, 0.3 Na_2_-GTP, and 7 K_2_-phosphocreatine, pH 7.3, with KOH. Series resistance was monitored and compensated online using the bridge-balance circuit of the amplifier. Experiments were discarded if the initial RMP was more depolarized than –60 mV, or if series resistance rose above 30 MΩ, or if there were fluctuations in temperature during the course of the experiment. Voltages have not been corrected for the liquid junction potential, which was experimentally measured to be ~8 mV.

#### STA and data analysis.

Physiologically relevant measurements from electrophysiological recordings (Fig. 1) were computed, employing previously established procedures (Ashhad et al. 2015; Ashhad and Narayanan 2016; Johnston and Narayanan 2008; Narayanan and Johnston 2007; Rathour et al. 2016). Input resistance (*R*_in_) was measured from a current-voltage curve constructed from the steady-state response of neurons to pulse-current injections (amplitude –50 to +50 pA; Fig. 1*A*). This current-voltage curve was fitted with a straight line whose slope defined *R*_in_ (Fig. 1*A*). A linear chirp current (Fig. 1*B*) spanning 15 Hz in 15 s was employed to estimate local impedance. To obtain the impedance amplitude profile (ZAP), the Fourier transform of the neuronal voltage response to the chirp current was divided by the Fourier transform of the chirp current. The frequency at which this ZAP reached its maximum (|*Z*|_max_) formed the resonance frequency (*f*_R_), and resonance strength (*Q_R_*) was defined as |*Z*|_max_/|*Z* (0.5 Hz)| (Fig. 1*C*). Sag was defined as the percentage difference in the peak to the steady-state response of the neuron to a –100-pA current injection. α-Excitatory postsynaptic potentials (α-EPSPs) were measured for current injections of the form *I*_α_ = *I*_max_ *t* exp (–α*t*), with α = 0.1/ms, where *I*_max_ governs maximum current and *t* is time. Temporal summation (*S*_α_) was computed as the ratio of the fifth α-EPSP amplitude to the first α-EPSP amplitude from the voltage response to five α-excitatory postsynaptic current injections at 20 Hz (Fig. 1*E*). The firing rate profile of these neurons in response to positive current pulses (0- to 250-pA amplitude) for a duration of 700 ms was also measured (Fig. 1*D*).

For measuring STA, zero mean GWN with the standard deviation (σ_noise_: 0.5–2 nA) adjusted to obtain ~1 Hz firing in the neuron was injected into the cell (Agüera y Arcas and Fairhall 2003; Badel et al. 2008; Bryant and Segundo 1976; Das et al. 2017; Das and Narayanan 2014, 2015; Dayan and Abbott 2005; Eggermont et al. 1983; Famulare and Fairhall 2010; Haas et al. 2007; Mainen and Sejnowski 1995; Prescott et al. 2008a; Ratté et al. 2013; Rieke et al. 1999; Schwartz et al. 2006). Neuronal voltage response to the GWN was measured (sampling rate: 20 kHz) for sufficient sweeps to harvest sufficient spikes (~1,000) toward computing the STA (Fig. 1, *F* and *G*). In each sweep, the current injection was designed such that the GWN (duration: 12 s) was preceded by a 250-ms pulse of –100 pA, followed by a duration of zero current for 500 ms before the start of the GWN so as to assess changes in series resistance, RMP, or other instability in the recording during each sweep throughout the total experiment (total duration of sweep ~14 s). Given the requirement of independent spikes for computing STA and the low average firing rate maintained therein, sweeps were repeated multiple times (~50–60), interspersed by 10-s periods of no current injection to allow the cell’s membrane potential to settle back to resting potential before the next GWN sweep. This resulted in the total duration of the experiment for computing a single STA to be around 20 min. STA was calculated by averaging 500 ms of the GWN stimulus that preceded each spike in the recorded voltage trace spanning the time period of the GWN injection. The STA was smoothed using median smoothing over bins of 20 points (1 ms) for representational purposes and for computing measurements that were derived from the STA.

Experiments that required the assessment of the adaptability of STA to membrane voltage (see Fig. 3) or σ_noise_ (see Fig. 4) and experiments that tested the role of HCN channels on STA measurements (see Figs. 5 and 6) required two distinct measurements of STA. As this experimental design entailed long-term recordings, it was essential to ensure that STA measurements were not significantly changing as a function of time during the recording period. To do this, we performed a set of experiments in which the neuronal response to the same GWN stimulus was measured twice (each measurement period spanning around 20 min) from the same neuron and STA measurements from these two durations were compared to ask if there were significant time-dependent changes in STA and other intrinsic measurements. In experiments that were designed to test the adaptability of STA to the membrane potential, the neuronal response to GWN stimulus of the same σ_noise_ was recorded for the same neuron at –70 mV and –60 mV (see Fig. 3). For determining STA adaptability to input statistics, the procedure was repeated for two different values of σ_noise_ in the same cell adjusted to obtain ~1 Hz and ~5 Hz firing rate, respectively, per cell (see Fig. 4), measured at the same membrane voltage. In experiments in which HCN channels were pharmacologically blocked (see Figs. 5 and 6), 20 µM 4-ethylphenylamino-1,2-dimethyl-6-methylaminopyrimidinium chloride (ZD7288; Tocris Bioscience), an irreversible blocker of HCN channels (Gasparini and DiFrancesco 1997; Narayanan and Johnston 2007, 2008; Shah et al. 2004), was perfused through the extracellular bath solution after an initial measurement of STA in the absence of ZD7288. Neuronal response to GWN stimuli was recorded with and without ZD7288 at the same membrane potential and same σ_noise_ to avoid confounds due to voltage and input dependence of STA. Median smoothing was done over 60 points (3 ms) for all traces (pretreatment, posttreatment, and control) for these sets of experiments to account for the larger fluctuations observed in the STA traces post-ZD7288 treatment (see Fig. 5*A*).

STA measurements (Fig. 1, *G* and *H*) were computed as described previously (Das and Narayanan 2014, 2015). The positive peak of the STA, $I_{\text{STA}}^{\text{peak}}$, was calculated as a metric of neuronal excitability. Specifically, a low value of $I_{\text{STA}}^{\text{peak}}$ would indicate that neuronal spike could, on the average, be elicited with a small current injection, implying higher excitability of the neuron under consideration. We employed two distinct STA-dependent measures of coincidence detection, which were derived and compared with more standard metrics of coincidence detection earlier (Das and Narayanan 2015). The total CDW (*T*_TCDW_) was measured as the period between spike occurrence and the time point at which the STA crossed the zero line, and the effective CDW (*T*_ECDW_) was computed from the spike-proximal positive lobe (SPPL; Fig. 1*G*) as:

(1) $$T_{\text{ECDW}}=\sqrt{\frac{\int_{-T_{\text{TCDW}}}^{0}t^{2}\;{\text{STA}}^{2}(t)dt}{\int_{-T_{\text{TCDW}}}^{0}{\text{STA}}^{2}(t)dt}}$$

The above definition of *T*_ECDW_ was developed (Das and Narayanan 2015) to account for the asymmetric positive weightage given to inputs arriving within the total CDW (note that the shape of the STA displays a sharp decay to zero; e.g., Fig. 1*G*).

To quantify the spectral characteristics of the STA, we computed the Fourier transform of the STA and measured STA characteristic frequency (*f*_STA_) as the frequency at which the |STA(*f*)| amplitude was maximum. The strength (*Q*_STA_) of this spectral selectivity was defined as |STA(*f*_STA_)|/|STA(0.5 Hz)|. Wavelet analysis was performed using the complex Morlet wavelet, and wavelet coefficients were computed spanning a frequency range of 1–85 Hz to assess the temporal location of the spectral selectivity in the STA (Fig. 1*I*). This allowed us to jointly visualize the relationship between the frequency selectivity in the STA and its temporal structure.

All data analyses were performed using custom-written software in Igor Pro (Wavemetrics), and statistical analyses were performed using the R computing package (http://www.r-project.org/). To depict the variability that was inherent in the measurements, all measured data points are reported rather than presenting only their statistics (Marder and Taylor 2011).

## RESULTS

### 

#### Theta-frequency spectral selectivity and gamma-range CDW in hippocampal STA.

**Fig. 2.**
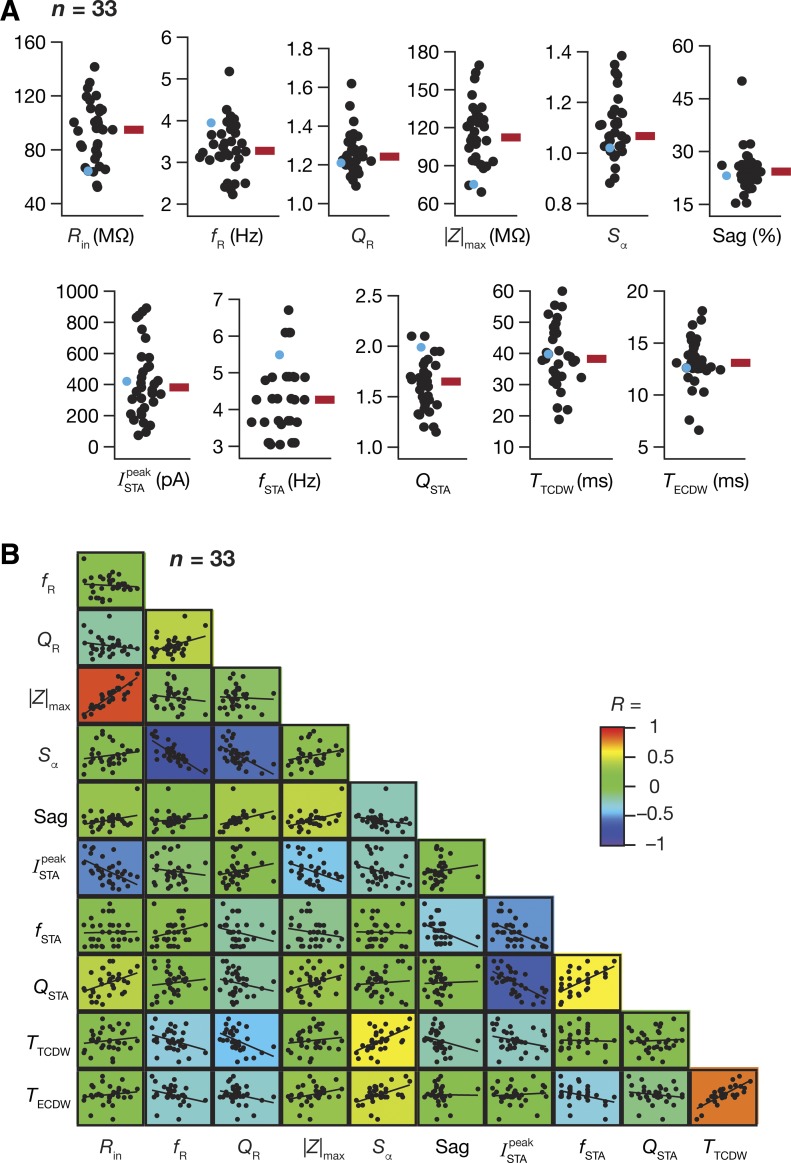
Hippocampal pyramidal neurons exhibit theta-frequency selectivity and gamma-range coincidence detection in their STA with differential correlations across different intrinsic measurements. *A*: population distribution (*n* = 33) for all 11 measurements shown with their respective median values (red rectangle). The cyan circles show the corresponding measurement for the example neuron depicted in Fig. 1. *B*. pairwise scatterplots with respective linear fits across all 11 intrinsic measures are overlaid on the lower diagonal elements of a color-coded matrix containing the respective Pearson’s correlation coefficient value (*R*).

We measured 11 different intrinsic physiological properties (at RMP) spanning neuronal excitability and frequency selectivity, including 5 measures derived from the STA, from hippocampal pyramidal neurons (*n* = 33 cells) in the presence of synaptic blockers (Figs. 1 and 2). The STA manifested theta-frequency selectivity (median *f*_STA_ ~ 4.5 Hz) with a strong selectivity strength (median *Q*_STA_ ~ 1.7) and was observed in conjunction with the well-established subthreshold theta-frequency resonance (Fig. 2*A*). Whereas the total CDW was more reflective of the membrane time constant of these neurons (median *T*_TCDW_ ~ 40 ms), the effective CDW, which accounts for the shape of the STA, was in the gamma-frequency range (median *T*_ECDW_ ~ 13 ms). These electrophysiologically measured values quantitatively matched their counterparts in our computational model with reference to the specific somatic values of $I_{\text{STA}}^{\text{peak}}$, *f*_STA_, *Q*_STA_, *T*_TCDW_, and *T*_ECDW_, thereby validating the predictions on theta-frequency selectivity and gamma-range coincidence detection in hippocampal somatic STA. Specifically, prior modeling results corresponding to hippocampal somatic STA (Fig. 7 of Das and Narayanan 2015) had predicted $I_{\text{STA}}^{\text{peak}}$ to be ~200 pA (experimental range in Fig. 2*A*: 50–900 pA), *f*_STA_ to be in the theta-range ~5 Hz (Fig. 2*A*: 3–7 Hz), *Q*_STA_ to be ~1.2 (Fig. 2*A*: 1.2–2.2), *T*_TCDW_ to be ~45 ms (Fig. 2*A*: 20–60 ms), and *T*_ECDW_ to be ~11 ms (Fig. 2*A*: 6–18 ms), which are within the ranges obtained with our current electrophysiological measurements.

To assess correlations between the STA measures and other established intrinsic measures, we plotted a correlation matrix, which was constructed by computing pairwise correlations between all of the 11 measurements across the 33 recordings (Fig. 2*B*). Expectedly, established measures of intrinsic excitability (*R*_in_ and |*Z*|_max_) were positively correlated, and measures that are known to be critically reliant on HCN channels (*S*_α_, *f*_R_, Sag, and *Q*_R_) also showed strong positive or negative correlations. With reference to the STA measurements, the $I_{\text{STA}}^{\text{peak}}$was negatively correlated with the excitability measures (*R*_in_ and |*Z*|_max_), confirming our computational prediction (Das and Narayanan 2014, 2015) that the $I_{\text{STA}}^{\text{peak}}$ was an effective measure of neuronal excitability, with a larger $I_{\text{STA}}^{\text{peak}}$ indicative of lower excitability.

The total and effective CDW showed strong negative correlations with *f*_R_ and *Q*_R_, suggesting that strongly resonant neurons also exhibit narrower windows for detection of coincident excitatory inputs. We noted this to be in congruence with the predicted relationship between the class of STA and the encoding strategy of the neuron. Specifically, neurons endowed with class II/III STA, characterized by a narrow SPPL and a negative lobe preceding the SPPL, are said to serve as better coincidence detectors and synchrony decoders because of their ability to respond to precisely timed inputs occurring within narrow temporal windows (Ratté et al. 2013). Our measures of CDW derived from the STA proffer a quantitative description of this prediction, supported also by the fact that a strong, positive correlation was observed between the CDWs and the summation ratio of α-EPSPs (*S*_α_) with larger CDW correlated with a high *S*_α_, a physiological signature of an integrator type neuron. Additionally, we found that measurements reliant on HCN channel expression, such as *f*_R_, *Q*_R_, Sag, and *S*_α_, showed strong correlations with one or both CDW measurements. Together, these observations suggest that cells endowed with higher HCN channel expression could detect coincident inputs over smaller time windows, specifically in the gamma-range. Importantly, although *f*_STA_ and *Q*_STA_ were strongly correlated, there was weak, albeit positive, pairwise correlation between *f*_STA_ and *f*_R_, confirming the dissociation between these two forms of frequency selectivity (Das and Narayanan 2015). This is to be expected owing to differential dependencies of *f*_STA_ and *f*_R_ on different neuronal parameters (Das and Narayanan 2015), and because *f*_STA_ is also dependent on input statistics (see below), a parameter that cannot be controlled for the measurement of *f*_R_.

#### Neuronal spectral selectivity and CDW are dependent on the membrane potential.

**Fig. 3.**
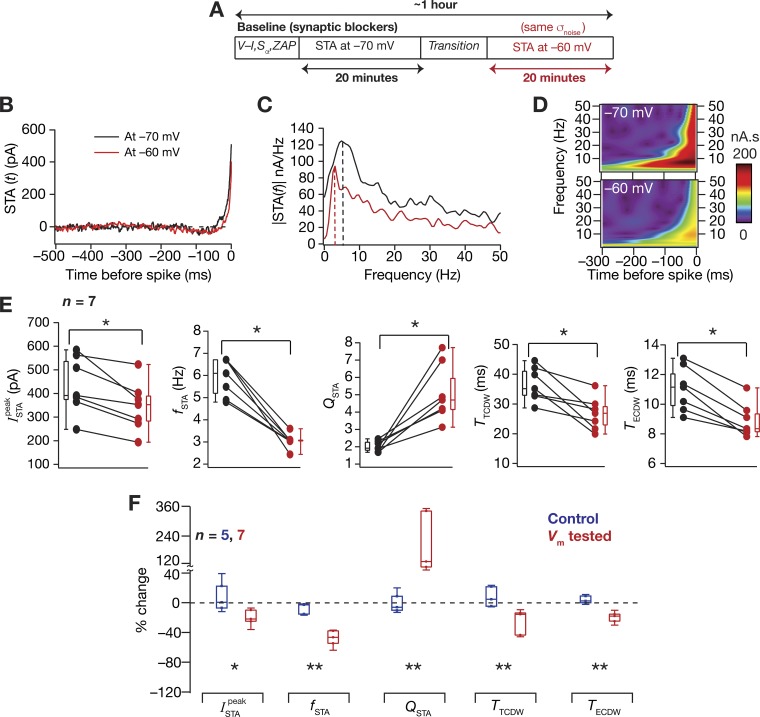
Hippocampal STA was dependent on neuronal membrane potential. *A*: the experimental protocol for measuring the dependency of STA on neuronal membrane potentials. *B* and *C*: the STA (*B*) and its Fourier (*C*) and wavelet (*D*) transform magnitudes measured in the same neuron at –60 mV (red) and –70 mV (black). *E*: significant effect (Wilcoxon signed-rank sum test, *P < 0.05, *n* = 7) of altering membrane potential was seen on $I_{\text{STA}}^{\text{peak}}$, *f*_STA_, *Q*_STA_, *T*_TCDW_, and *T*_ECDW_. For these panels, black and red represent measurements at –70 mV and –60 mV, respectively. *F*: percent changes in all of the STA measurements obtained on depolarizing the cell (red, membrane potential tested) are plotted showing the variable impact of changing membrane potential on each measurement. Comparisons with control experiments (blue, control) are shown, which involved measuring the STA twice, with a temporal separation similar to the other experiments, at the same voltage and with the same GWN (Mann-Whitney *U*-test, *P < 0.05, **P < 0.01).

A large number of intrinsic neuronal properties are voltage dependent, defined by the gating kinetics of ion channels that mediate and regulate them and by the interactions among the several coexpressing ion channels. As a direct consequence of the mediating role of HCN channels, subthreshold *f*_R_ and *Q_R_* increase with hyperpolarization of membrane voltages (Hu et al. 2002; Narayanan et al. 2010; Narayanan and Johnston 2007). Given the expression of theta-frequency selectivity in the STA, we asked ourselves how the *f*_STA_ and related measures are altered by the RMP. Our previous computational models have predicted a significant voltage dependence of theta-frequency selectivity in the STA and the CDWs, with different channels differentially contributing to such voltage dependence (Das and Narayanan 2014, 2015). To electrophysiologically assess the voltage dependence of STA spectral selectivity and CDW, we measured STA from individual neurons at –60 mV and at –70 mV while keeping σ_noise_ the same for both membrane voltages. In implementing this, the standard deviation was first adjusted to obtain ~1-Hz firing at –70 mV, and the same GWN input was used at –60 mV. We found that depolarization of membrane potential resulted in significant reductions in $I_{\text{STA}}^{\text{peak}}$, *f*_STA_, *T*_TCDW_, and *T*_ECDW_ and significantly increased *Q*_STA_ (Fig. 3, *A–E*). We reasoned that these effects were a consequence of *1*) the neuronal state being closer to action potential threshold when it was depolarized, thereby reducing $I_{\text{STA}}^{\text{peak}}$, with perithreshold conductances significantly contributing to increased *Q*_STA_ and reduced CDW values; and *2*) the depolarization-induced deactivation of HCN channels contributing to the reduction of *f*_STA_ (Das and Narayanan 2014).

While the decrease in *f*_STA_ with depolarization is consistent with corresponding changes in *f*_R_ reported in literature, the increase in *Q*_STA_ is contradictory to changes in *Q*_R_ (Narayanan and Johnston 2007; Rathour and Narayanan 2012). This difference could be attributed to the contribution of spiking conductances, namely sodium and delayed-rectifier potassium channels, toward regulating the STA by dictating both the spike initiation dynamics, as well as repolarization kinetics (Das and Narayanan 2014; Prescott et al. 2006). Although subthreshold *Q_R_* is primarily unaffected by these spike-generating conductances that are activated by suprathreshold voltages, the emergence of spectral selectivity in the STA would critically depend on these conductances, which would determine how “effectively” a neuron can fire in response to its preferred input. The dissociation between the voltage dependence of *f*_STA_ and *Q*_STA_ is thus indicative of an important neuronal feature, where different channels could differentially contribute to individual measurements in a state-dependent manner (Das et al. 2017; Das and Narayanan 2014, 2015; Hu et al. 2009). We also noted that this dissociation occurred, despite a strong correlation between *f*_STA_ and *Q*_STA_ at RMP (Fig. 2*B*).

#### Neuronal spectral selectivity adapt to input statistics.

**Fig. 4.**
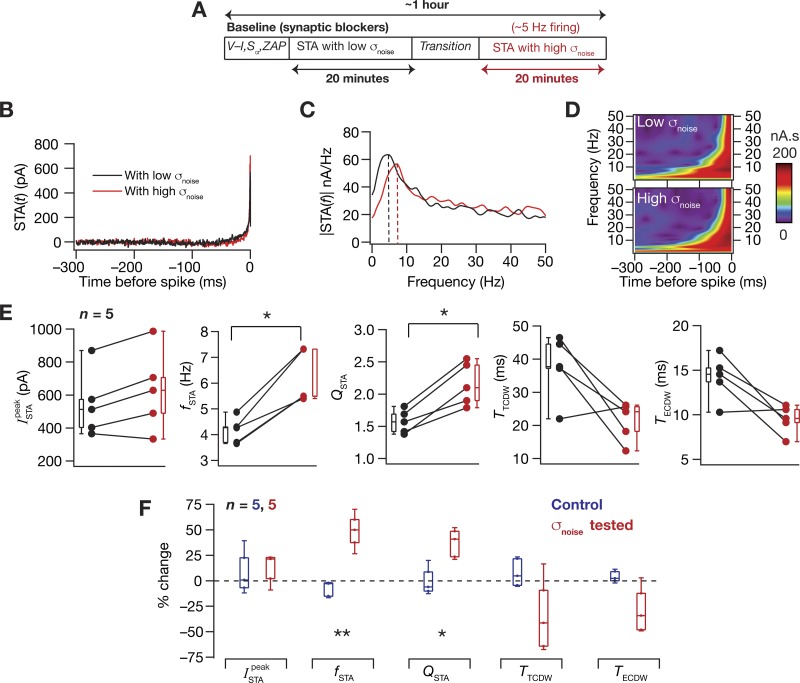
Hippocampal STA was dependent on the input statistics. *A*: the experimental protocol for measuring the dependency of STA on input statistics by measuring STA with GWN of two different σ_noise_ values. *B* and *C*: the STA (*B*) and its Fourier (*C*) and wavelet (*D*) transform magnitudes measured in the same cell at resting membrane potentials with two different values of σ_noise_, the standard deviation of the GWN. *D*: significant effect (Wilcoxon signed-rank sum test, *P < 0.05, *n* = 5) of altering membrane potential was seen on *f*_STA_ and *Q*_STA_. For these panels, black and red represent measurements with low (adjusted to obtain ~1-Hz average firing rate) and high (adjusted to obtain ~5-Hz average firing rate) σ_noise_, respectively. *F*: percent changes in all of the STA measurements (red, σ_noise_ tested) obtained on altering the σ_noise_ are plotted showing the variable impact of altering σ_noise_ on each measurement. Comparisons with control experiments (blue, control) are shown, which involved measuring the STA twice, with a temporal separation similar to the other experiments, at the same voltage (RMP) and with the same GWN (Mann-Whitney *U*-test, *P < 0.05, **P < 0.01).

Prior literature has underscored the adaptability of neuronal STA to stimulus statistics (Famulare and Fairhall 2010; Mainen and Sejnowski 1995), with quantitative predictions for increases in somatic $I_{\text{STA}}^{\text{peak}}$, *f*_STA_, and *Q*_STA_ and decreases in *T*_TCDW_ and *T*_ECDW_, with increase in stimulus variance (Fig. 7 in Das and Narayanan 2015). Additionally, given the correlations, or lack of therein, between the STA measures and related intrinsic properties (Fig. 2*B*), it was imperative to quantitatively assess the impact of the GWN standard deviation used in our studies on the somatic STA to understand the adaptability of these measurements with changes in input statistics. Therefore, we electrophysiologically tested our quantitative predictions by measuring STA at neuronal RMP with two different values of σ_noise_, the lower one adjusted to elicit ~1-Hz firing and the higher one for ~5-Hz firing in the same neuron. We compared STA measurements computed with these two input stimuli that were different in their variance and found conclusions from these experiments agreed with previous quantitative predictions. Specifically, with increase in σ_noise_, there were increases in *f*_STA_ and *Q*_STA_, coupled with a modest constriction of the SPPL (Fig. 4), as predicted by our simulations (Das and Narayanan 2014, 2015). Although there was a concomitant increase in $I_{\text{STA}}^{\text{peak}}$ coupled to a reduction in both CDW measures in most cells, these changes were not statistically significant (Fig. 4, *E* and *F*). In summary, neuronal spectral selectivity and coincidence detection capabilities were critically dependent on the state of neuronal membrane potential and were adaptable to the statistics of neuronal input.

#### Theta-frequency selectivity in STA is mediated by HCN channels.

**Fig. 5.**
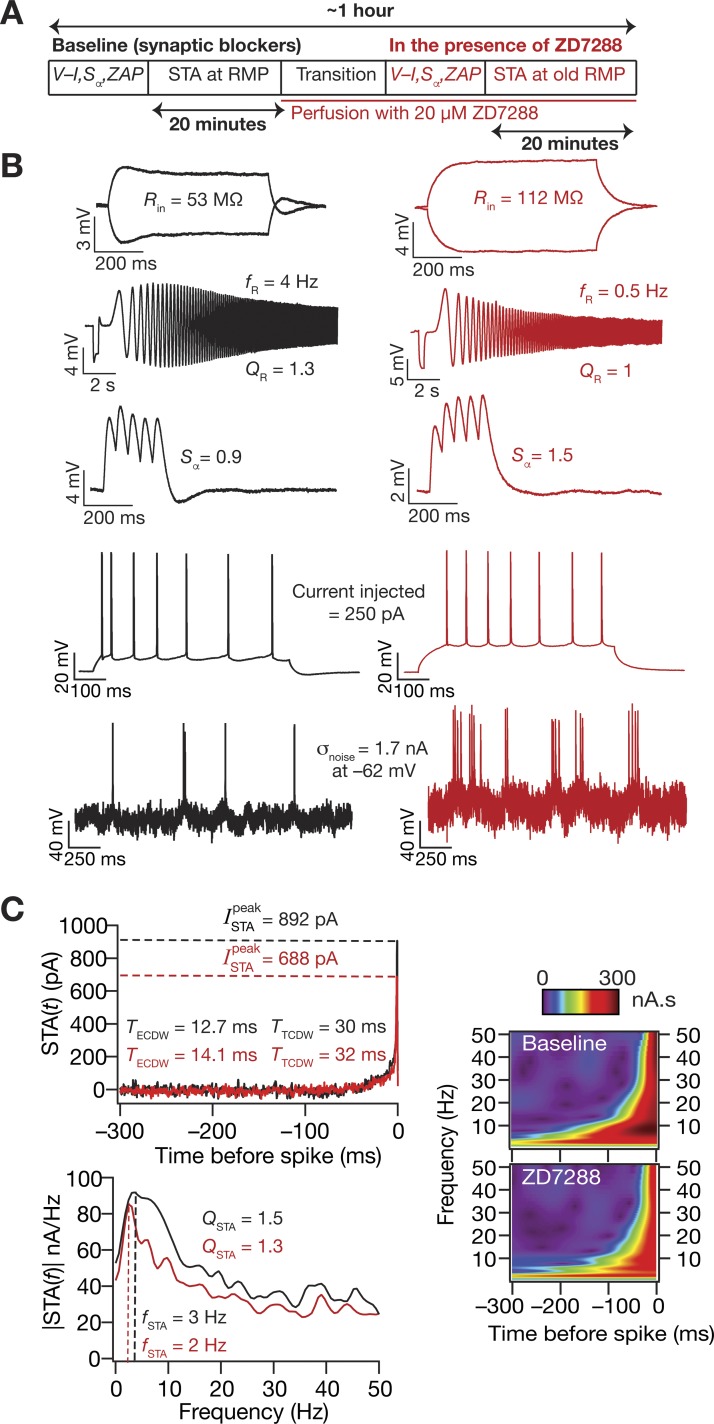
Traces from a typical experiment involving the measurement of STA and other intrinsic properties before and after pharmacological blockade of HCN channels. *A*: protocol employed for assessing the role of HCN channels on neuronal STA using HCN channel blocker ZD7288 (20 µM). *B*: all measurements were performed both before and after perfusion of ZD7288 through the bath. Example traces are shown for measuring input resistance, resonance frequency, and temporal summation obtained before (black) and after (red) blocking HCN channels. *C*: STA (*top*) and its Fourier transform magnitude (*bottom*) measured before and after ZD7288 application. Wavelet analysis of the STA showed a marked reduction in coefficients corresponding to theta-frequencies post-ZD7288 treatment (*left*).

**Fig. 6.**
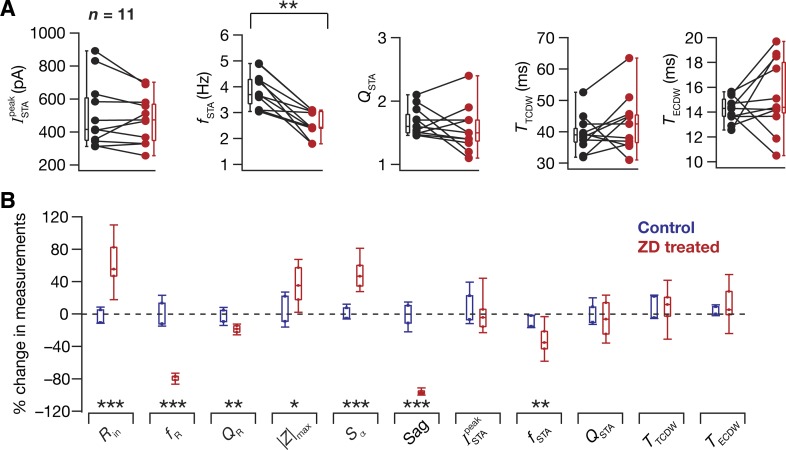
Pharmacological blockade of HCN channels reduced the STA frequency selectivity from the theta-frequency range to the delta-frequency range. *A*: plots of STA measurements before (black) and after (red) treatment with ZD7288. The protocol for all experiments in this panel was identical to the one shown in Fig. 5*A*. Statistically significant reduction in *f*_STA_ from the theta- to delta-frequency range was observed (Wilcoxon signed-rank sum test, **P < 0.05, *n* = 11), whereas no significant effect of treatment was seen on $I_{\text{STA}}^{\text{peak}}$, *Q*_STA_, and the CDWs. *B*: percent changes in all 11 measurements after the application of ZD7288, plotted as medians and their quartiles (red, ZD treated), compared with control (blue, control) experiments, which involved an identical protocol as Fig. 5*A*, except without the bath application of ZD7288 (Mann-Whitney *U*-test, *P < 0.05, **P < 0.01, ***P < 0.001).

We had earlier predicted using computational analyses that, in the absence of HCN channels, hippocampal somatic STA spectral selectivity would switch from the theta- to the delta-frequency range (0.5–4 Hz) (Das and Narayanan 2014, 2015). To test this, and motivated by the significant correlations (Fig. 2*B*) across baseline electrophysiological measurements that are known to critically depend on HCN channels in hippocampal pyramidal neurons, we measured STA from the same cell at the same membrane voltage using the same σ_noise_ before and after application of an HCN-channel blocker ZD7288 (Fig. 5*A*). This experimental design was implemented to avoid potential confounds from the dependence of STA measurements on membrane voltage and on neuronal input statistics (Figs. 3 and 4). Concordant with computational predictions, we found that *f*_STA_ reduced significantly to the delta-frequency range after blockade of HCN channels (Figs. 5*C* and 6). We noted that this delta-frequency selectivity in the STA did not emerge from corresponding subthreshold resonance because the ZAP manifested low-pass characteristics bereft of any frequency tuning in the presence of ZD7288 (Fig. 5*A*), setting the *f*_R_ to be < 0.5 Hz in all cases. These observations confirmed computational predictions on the emergence of STA spectral selectivity, even in the absence of subthreshold resonance, furthering the dissociation between these two forms of spectral selectivity (Das and Narayanan 2014, 2015). Statistical analyses revealed no significant changes in $I_{\text{STA}}^{\text{peak}}$, *Q*_STA_, or the CDWs after blockade of HCN channels (Fig. 6). Together, these results clearly demonstrated that theta-frequency selectivity in the somatic STA of CA1 pyramidal neurons was critically dependent on the presence of HCN channels.

## DISCUSSION

In this study, we have positively tested several predictions from our previous computational studies, primarily showing that CA1 pyramidal neurons exhibit theta-frequency selectivity in their STA, and that blocking HCN channels significantly reduces the frequency of this selectivity to the delta-range. These results further expand the roles of HCN channels to theta-frequency selectivity in the STA, in addition to established roles in regulating neuronal excitability, synaptic plasticity rules, temporal summation, subthreshold resonance, and intrinsic phase responses (Das et al. 2017; Honnuraiah and Narayanan 2013; Hutcheon and Yarom 2000; Johnston and Narayanan 2008; Magee 1998, 1999a; Narayanan and Johnston 207, 20010; Sinha and Narayanan 2015). Although the role of HCN channels in regulating STA has been electrophysiologically assessed in other cell types (Haas et al. 2007), our study establishes a clear role for these channels in CA1 pyramidal neuron STA, apart from unveiling specific roles for these channels in spectral selectivity and coincidence detection using STA-derived metrics.

### 

#### Theta-frequency selectivity in the STA.

The role of HCN channels in mediating theta-frequency selectivity in the STA, apart from adding to the repertoire of functions of HCN channels in hippocampal pyramidal neurons, also unearths critical relationships between subthreshold and suprathreshold frequency selectivity in these neurons. Our previous computational studies had demonstrated that, when HCN channel conductance is the only subthreshold conductance in the model (apart from the spike-generating conductances), the STA characteristic frequency (*f*_STA_) is positively correlated to the subthreshold *f*_R_ of the model neuron (Das and Narayanan 2014). However, the addition of other subthreshold conductances to the model, specifically those of T-type calcium and A-type potassium channels, led to a significant dissociation between the two frequency selectivity measures (Das and Narayanan 2015). In addition, when HCN-channel conductances were removed from the model, although subthreshold resonance was completely abolished, spectral selectivity in the STA was observed, with *f*_STA_ dropping to the delta-frequency range from the theta-frequency range (which was observed when HCN channel conductances were present in the model). This observation where delta-frequency selectivity in the STA emerges, despite the absence of subthreshold resonance constitutes another form of dissociation between these two forms of spectral selectivity (Das et al. 2017; Das and Narayanan 2014, 2015).

Our present electrophysiological characterization of CA1 pyramidal neurons confirms the dissociation between these forms of selectivity, whereby *1*) there is a lack of significant correlation between *f*_R_ and *f*_STA_ across the different recorded neurons (Fig. 2), and *2*) when HCN channels are blocked, delta-frequency selectivity in the STA emerges, despite the absence of subthreshold resonance (Figs. 5 and 6). We had previously demonstrated in models that the spike-generating conductances (transient sodium and delayed rectifier potassium) of the CA1 pyramidal neuron mediate delta-frequency spectral selectivity in the absence of HCN channels (Das and Narayanan 2014). Although these conductances have negligible effects on the subthreshold *f*_R_, our modeling predictions demonstrated these to be capable of mediating delta-frequency selectivity in the STA. Although our electrophysiological characterization confirms observations on the emergence of delta-frequency selectivity in the absence of HCN channels, the computationally driven postulate on the role of spike-generating conductances as the specific mediators of this delta-frequency selectivity needs electrophysiological verification. One way to test this postulate would be to measure STA, in the presence of ZD7288, with various levels of partial blockade of either the transient sodium and delayed rectifier potassium channels. A complete blockade of these spike-generating conductances would be infeasible because spike generation would be hampered in their absence. Future studies could also pharmacologically assess the role of other ion channels, such as the T-type calcium channel and A-type potassium channel, which are known to be expressed abundantly in CA1 pyramidal neurons and to regulate subthreshold resonance (Hoffman et al. 1997; Magee and Johnston 1995; Migliore et al. 1999; Migliore and Shepherd 2002; Rathour et al. 2016; Rathour and Narayanan 2012) and spectral selectivity in the STA of these neurons (Das et al. 2017; Das and Narayanan 2015).

#### Gamma-range coincidence detection in the STA.

Apart from theta-frequency selectivity in the STA, we also showed that hippocampal neurons are endowed with gamma-range CDWs, which enable these neurons to decode synchronous gamma-frequency inputs. This ability to function as a gamma-range coincidence detector is an important feat for CA1 pyramidal neurons, especially with reference to the rate-temporal coding debate, for achieving enhanced spike-phase coherence with reference to a LFP oscillation, for encoding of specific spatial as well as nonspatial information, and for allowing different neuronal assemblies to route information selectively (Buzsáki 2010; Colgin et al. 2009; Das et al. 2017; Das and Narayanan 2014, 2015; Jaramillo and Kempter 2017; Lisman and Jensen 2013; Mehta et al. 2002; Sinha and Narayanan 2015; Terada et al. 2017; Zheng et al. 2016a, 2016b). The biophysical mechanisms of coincidence detection have been studied in other systems. There, coincidence detection has been found to be critically reliant on the morphology of the neurons and on the expression profile of ion channels, including HCN and low-voltage activated potassium channels, that regulate the subthreshold integrative properties and perithreshold membrane dynamics (Goldberg and Brown 1969; Joris et al. 1998; Khurana et al. 2012; Mathews et al. 2010; Schaefer et al. 2003; Softky 1994). Given the evidence for differential gamma-frequency coherence in different hippocampal subfields and the anatomical architecture of the hippocampus, it is imperative that CA1 neurons demonstrate coincidence detection ability in the gamma-frequency range (Bieri et al. 2014; Buzsáki 2010; Colgin et al. 2009; Fernández-Ruiz et al. 2017). Our measurement of CDW from the STA implies that CA1 neurons are intrinsically tuned to elicit action potentials in response to gamma-frequency inputs. We had systematically assessed the quantitative equivalence of the CDW measurements to other measurements of coincidence detection earlier (Das and Narayanan 2015). Together, the electrophysiological demonstration of gamma-range CDW in hippocampal neurons, and the dependence of this CDW on input statistics and membrane voltage, strengthens the argument that these neurons are endowed with the machinery to process gamma-frequency inputs (Das and Narayanan 2015).

Although our computational studies predicted a dependence of the CDW on HCN channel expression (Das and Narayanan 2015), and although the CDW measures were negatively correlated with subthreshold *f*_R_ under baseline conditions (Fig. 2), we did not observe a significant change in CDW (or *Q*_STA_, of which the computational studies predicted a dependence) on blockade of HCN channels with ZD7288. This could be a result of multiple factors regulating the CDW, including interactions among other ion channels (Goldman et al. 2001; Marder and Taylor 2011) that express in hippocampal pyramidal neurons, with such interactions also reliant on the specific voltage at which the CDW was measured (Das and Narayanan 2015; Khurana et al. 2012). In addition, as the computational models were not endowed with all of the channels expressing in CA1 pyramidal neurons, one possibility is that these results are an outcome of interactions among other channels (besides the ones included in the model) that play important roles in the regulation of STA and its measurements (Das and Narayanan 2015).

A third possibility is the enhanced excitability associated with the blockade of HCN channels (Figs. 5 and 6), which resulted in larger deflections for the same GWN current stimulus when HCN channels were blocked (Fig. 5*B*). Although we had employed the same σ_noise_ before and after the blockade of HCN channels, this ZD7288-induced enhancement in excitability is equivalent to enhancing the σ_noise_. With an increase in σ_noise_, we had observed significant reductions in the CDW measurements (Fig. 4). Therefore, it is possible that there are at least two opposing changes in play: one owing to the blockade of HCN channels, possibly leading to an increase in CDW (Das and Narayanan, 2014), and another resulting in a larger deflection owing to higher excitability, which results in a reduction of CDW (Fig. 4). Therefore, it is possible that these opposing changes resulted in some cells in which there was a reduction in CDW, whereas in others there was an increase, together leading to a statistically nonsignificant change in CDW after treatment with ZD7288. However, with reference to *f*_STA_, we noted that there was a significant reduction in the delta-frequency (Figs. 5 and 6), despite the possible presence of a opposing force driven by the larger fluctuations (Fig. 4). Therefore, this enhancement in response fluctuations (which could also be partly due to channels that were absent in the computational model) could play a role in the discrepancies observed in our computational and electrophysiological conclusions. Finally, although nonspecificities of ZD7288 with reference to synaptic transmission (Chen 2004; Chevaleyre and Castillo 2002) might not affect our measurements, because all recordings were performed in the presence of synaptic blockers, there are other nonspecificities (Sánchez-Alonso et al. 2008) that might act as potential confounds.

#### Channel interactions, heterogeneities, and degeneracy in the STA.

We demonstrated significant correlations and dissociations between different intrinsic measurements under baseline conditions (Fig. 2), and between changes in these intrinsic measurements introduced by alterations to membrane potential (Fig. 3), to stimulus statistics (Fig. 4) and to channel composition (Figs. 5 and 6). These results, in conjunction with prior computational and experimental analyses in CA1 pyramidal neurons (Das et al. 2017; Das and Narayanan 2014, 2015; Rathour et al. 2016; Rathour and Narayanan 2014; Ratté et al. 2013), underscore the critical role of interactions among different ion channels in regulating neuronal physiological measurements. Our study also reports significant heterogeneities in STA measurements, apart from confirming previously reported heterogeneities in other intrinsic properties. Specifically, the baseline intrinsic measurements (Fig. 2) and the quantitative impact of the alterations to membrane voltage (Fig. 3), input statistics (Fig. 4), and channel composition (Figs. 5 and 6) demonstrate significant neuron-to-neuron variability. This variability was observed, despite our specific focus on the somata of pyramidal neurons in the middle hippocampus along the dorsoventral axis and on the center of the proximal-distal axis of the hippocampus. There are numerous ramifications for such heterogeneity in neuronal intrinsic properties in general, and neuronal STA in particular (Grashow et al. 2010; Marder 2011; Marder et al. 2014; Marder and Goaillard 2006; Marder and Taylor 2011). Specifically, it has been shown that the presence of such heterogeneities in neuronal STA plays a critical role in de-correlating neuronal firing and in enhancing information encoding in mitral cells of the olfactory bulb (Padmanabhan and Urban 2010). Future studies in the hippocampus could assess the role of diversity in neuronal STA on information encoding and transfer across the hippocampal trisynaptic circuit, especially accounting for adult neurogenesis in the dentate gyrus and the recurrent circuitry in the CA3 (Anderson et al. 2007).

As mentioned above, for the purposes of this study, we have focused our recordings on the middle hippocampus along the dorsoventral axis, the central part of the proximal-distal axis, and did not distinguish between superficial and deep neurons. Although there are lines of evidence in the literature that intrinsic excitability (measured from *R*_in_ and firing rate profiles) of the somata along the superficial-deep axis are not significantly different from each other (Lee et al. 2014), we do not know if there are differences in spectral selectivity properties in the somata and dendrites of superficial vs. deep neurons. We also do not know if such differences would extend along the dorsoventral axis, where there have been reports of significant gradients in other intrinsic properties (Dougherty et al. 2012, 2013; Malik et al. 2016). Future studies should, therefore, systematically characterize gradients and local heterogeneities in spectral selectivity (subthreshold resonance and STA characteristics) and CDWs across the somatodendritic, dorsoventral, proximal-distal, and the deep-superficial axes of hippocampal neurons, including the potential role of morphological heterogeneities on these measurements (Danielson et al. 2016; Das et al. 2017; Dhupia et al. 2015; Dougherty et al. 2012, 2013; Jarsky et al. 2008; Lee et al. 2014; Malik et al. 2016; Maroso et al. 2016; Mizuseki et al. 2011; Thompson et al. 2008; Valero et al. 2015).

Finally, given the several dependencies of any given STA or CDW measurement on several channels and input characteristics, it is conceivable that the realization of a specific encoding system endowed with a given form of STA could be achieved with very disparate combinations of channel parameters and stimulus characteristics. In other words, conclusions from this study and from our previous computational studies (Das et al. 2017; Das and Narayanan 2014, 2015) clearly point to the expression of degeneracy (Edelman and Gally 2001) in the emergence of feature selectivity and coincidence detection in neuronal compartments. Future studies on STA and CDW measurements should, therefore, focus on the expression of degeneracy in the emergence of intraneuronal functional maps of these measurements by disparate channel conductances, and on the role of dendritic spikes in altering them (Das et al. 2017; Ermentrout et al. 2007; Kalmbach et al. 2017; Marder 2012; Narayanan and Johnston 2012; Rathour and Narayanan 2012; 2014; Ratté et al. 2013; Shah et al. 2010). These analyses should also be expanded to other neuronal subtypes to explore the possibility that different neuronal subtypes employ disparate strategies to achieve the same STA and CDW measurements. Such analyses would also reveal the channel expression and localization strategies employed by neurons in achieving analogous encoding and homeostasis capabilities in neuronal structures, also unveiling any potential correlations between channel expression profiles across the somatodendritic arbor (Gjorgjieva et al. 2016; Goaillard et al. 2009; Hanus and Schuman 2013; Nusser 2009, 2012; O’Leary et al. 2013, 2014; Rathour and Narayanan 2012, 2014; Schulz et al. 2006; Srikanth and Narayanan 2015; Vacher et al. 2008).

#### Plasticity and neuromodulation in the STA.

Neurons are plastic computational devices equipped with several forms of synaptic and intrinsic plasticity that alter channel/receptor expression profiles in these neurons, regulating their excitability, action potential firing patterns, intrinsic impedance profiles, and spectral response properties, and thereby adapting neuronal response to afferent inputs (Frick and Johnston 2005; Johnston and Narayanan 2008; Kim and Linden 2007; Mozzachiodi and Byrne 2010; Narayanan and Johnston 2007, 2008, 2012; Remy et al. 2010; Shah et al. 2010; Sinha and Narayanan 2015; Sjöström et al. 2008; Zhang and Linden 2003). In turn, plasticity in the synaptic and intrinsic neuronal properties can modify the rules for induction of neuronal plasticity (Anirudhan and Narayanan 2015; Ashhad and Narayanan 2013; Chen et al. 2006; Cooper and Bear 2012; Honnuraiah and Narayanan 2013; Luján et al. 2009; Narayanan and Johnston 2010; Nolan et al. 2004; Philpot et al. 2001, 2003; Sehgal et al. 2013; Shouval et al. 2002), thus evincing a complex consortium of factors that alter neuronal response properties, their spike initiation dynamics, and feature-selective gain modulation.

Our results pertaining to the STA in CA1 pyramidal neurons suggest multiple ion channels regulating different aspects of the spike initiation dynamics, with HCN channels mediating theta-frequency selectivity (Figs. 5 and 6) in the STA (Das and Narayanan 2014, 2015). Given the heavy coexpression of HCN channels with other subthreshold voltage-gated ion channels in the CA1 neurons and their spatiotemporal interactions therein, local or global plasticity in one or more of these ion channels could alter the STA, bringing about a transition in the operating mode of the neuron in terms of the class of excitability to which they belong, with important consequences for the function of the neuron in a network (Abouzeid and Ermentrout 2009; Das et al. 2017; Das and Narayanan 2014, 2015; Ermentrout 1996; Ermentrout et al. 2007; Golding and Oertel 2012; Khurana et al. 2011, 2012; Llinas et al. 2002; Mathews et al. 2010; Padmanabhan and Urban 2010; Prescott et al. 2008a; Ratté et al. 2013). Additionally, the demonstration of the heavy dependence of the STA on the membrane potential, as well as input statistics, implies that the conductance state of the neuron is a crucial regulator of the neuronal encoding strategy, translating to the STA being amenable to short-term adaptation, depending on the behavioral state of the animal (Destexhe et al. 2003; Mishra and Narayanan 2015; Prescott et al. 2006, 2008b).

In this context, neuromodulation is a crucial factor that defines and reflects the behavioral and motivational state of the animal, regulating ion channel physiology and the intrinsic properties of pyramidal neurons (Bargmann and Marder 2013; Lee and Dan 2012; Marder 2011, 2012; Marder et al. 2014; Marder and Thirumalai 2002). Specifically, HCN channel expression and kinetics are reliant on a multitude of signaling processes and, when combined with the differential expression of neuromodulatory receptors present in pyramidal neurons, allow for the possibility of a complex and nuanced tuning of the STA profile of these neurons with important ramifications for processing of synaptic inputs under physiological and pathophysiological conditions (Biel et al. 2009; Gasparini and Magee 2006; Haag and Borst 1996; Hansen and Manahan-Vaughan 2014; Hasselmo 1995; Kole et al. 2006; Lewis and Chetkovich 2011; Lörincz et al. 2002; McCormick and Pape 1990; Narayanan et al. 2010; Shah et al. 2010; Tanaka et al. 2012; Vacher et al. 2008; Werlen and Jones 2015). In addition to this, neuromodulation of other channels and receptors could also play a critical role in defining the specific characteristics of STA in CA1 pyramidal neurons. Thus the spike initiation dynamics and hence the encoding strategy employed by a neuron in a network are heavily amenable to significant alterations through neuromodulation, activity-dependent plasticity, and changes in input statistics, allowing for a state- and input-dependent transition in the operating mode of the neuron and possibly enabling it to adopt the most efficient encoding schema in a given behavioral scenario (Barlow 1961; Denève et al. 2017; Gallistel 2017; Narayanan and Johnston 2012; Simoncelli 2003; Simoncelli and Olshausen 2001; Stemmler and Koch 1999). Future experiments could focus on neuromodulation and activity-dependent plasticity of somatodendritic STA and CDW profiles and assess the impact of such adaptability to efficient coding within the neuron and across the network.

Together, our study establishes STA as a powerful tool to assess neuronal excitability, frequency selectivity, and CDWs in neuronal compartments. With specific reference to hippocampal physiology, we have demonstrated HCN channel-dependent theta-frequency selectivity in spike initiation dynamics and gamma-range CDWs in CA1 pyramidal neurons using novel measures derived from the STA. Our results also show critical regulation of the STA by the membrane potential and by input statistics, also revealing strong dissociations between the STA-based frequency selectivity and subthreshold impedance measurements in these neurons.

## GRANTS

This work was supported by the Wellcome Trust-DBT India Alliance (Senior fellowship to R. Narayanan; IA/S/16/2/502727), the Human Frontier Science Program Organization (R. Narayanan), the Department of Biotechnology (R. Narayanan), a Bristol Myers Squibb fellowship (A. Das) and the Ministry of Human Resource Development (A. Das and R. Narayanan).

## DISCLOSURES

No conflicts of interest, financial or otherwise, are declared by the authors.

## AUTHOR CONTRIBUTIONS

A.D. and R.N. conceived and designed research; A.D. performed experiments; A.D. analyzed data; A.D. and R.N. interpreted results of experiments; A.D. prepared figures; A.D. and R.N. drafted manuscript; A.D. and R.N. edited and revised manuscript; A.D. and R.N. approved final version of manuscript.
